# Treatment of Pyorrhea Alveolaris

**Published:** 1916-09

**Authors:** J. H. J. Upham


### TREATMENT OF PYORRHEA ALVEOLARIS.

BY DR. J. H. J. UPHAM.

I believe the condition is absolutely a surgical one, with medical treatment as an auxiliary aid.

There are two classical positive surgical principles in the treatment of suppuration anywhere in the body, where possible; first and foremost the free evacuation of the pus, second, the removal of local sources of irritation; with the modern addition of a third, the auxiliary constitutional treatment to raise the systemic resistance by the production of immunity, or remove any systemic predisposing cause.

Thus the first indication after the pus flow is established is surgical with all that that implies, including the evacuation of the pus, antiseptic applications, cleaning out the debris, the removal of deposits, the correction of mechanical sources of irritation whatever they may be. No matter what has been or may be discovered for the specific constitutional treatment, if it be infectious whether from the endamœba or what not, the opportunities for infection are evidently so omnipresent, that unless all such local conditions are corrected, re-infection is bound to occur.

Next, constitutional treatment; while the prepared specific treatment which I shall mention in a moment is still *sub-judice,* one should always look toward the correction also of any constitutional predisposing causes if present. Thus of course, diabetes, tuberculosis, neurasthenia and the like should have proper medical attention. I am not so sure as to the so-called rheumatic diathesis; there may be grounds for considering that a result rather than even a predisposing cause of the local condition.

The next is the specific treatment, and this has aroused a very great deal of interest. Acting on the hypothesis that the endamœba was the specific cause of pyorrhea, and having in mind the extraordinary amœbacidic action of ipecac and its active principle emetin, introduced by Rogers in 1912, Barrett and independently Bass and Johns began the use of this drug, and claim remarkable success. They reported the prompt disappearance of the amœba with extraordinary improvement in the local conditions.

The ordinary local treatment is applied as usual. The question of regeneration in the peridental tissues depends upon the extent to which they have been damaged. Certainly unless it occurs or until it occurs eternal vigilance must be exercised to prevent re-infection which otherwise is sure to occur, and often does occur anyway. Repeated examinations of the material from the pockets should be made from time to time and if endamœba reappears the emetin should be repeated. Realizing the very great likelihood of re-infection in spite of the greatest care, Bass and Johns advise repeating the three-days course of emetin anyway as a prophylactic, every three or four weeks, for two or three periods at least.

During the past year this treatment has had great publicity and probably a very wide trial. I do not think, however, always a fair trial. I say this because of the wide diversity of results apparently obtained. Some practitioners have written enthusiastically of the benefits, while others have minimized them or actually disparaged the use of the new remedy altogether. Evidently the personal equation is an active factor as is so often the case, and time alone with a more thorough working out of the exact technic will enable us to appraise this treatment at its true worth.

It seems to me, however, only fair to insist that one should not expect too much from the use of emetin; one has no right to expect miracles. We may have an analogous condition in the treatment of tuberculosis. Suppose that a specific is found which absolutely destroys the tubercle bacillus in the human body; in early cases of single infection this substance would promptly and effectively cure the patient. But given a case of mixed infection, of perhaps cavity formation full of broken down tissue, mixed with organisms of all sorts, would the specific cure such a condition? Not at all; undoubtedly it might help matters to some extent but the mixed infection still remains.

So in many cases of pyorrhea, in fact the most advanced ones, assuming for the sake of argument that the endamœba is the exciting cause, the use of emetin may remove the endamœba but leaves countless other organisms thoroughly capable of carrying on the morbid processes.

Therefore, I say that this new treatment to my mind has not had altogether a fair scientific trial, and is in danger of being discredited as a result. Some practitioners have given it as per directions on the package and omitted adjuvant treatment. Sometimes they get remarkable improvement, sometimes no benefit at all, and they become perplexed and dissatisfied.

Others, advanced operators, using thorough surgical or prophylactic treatment, try the emetin in conjunction with their present efficient methods and claim perhaps no marked benefit. This side of the question could only be answered after a longer trial. A careful compilation of cases would be necessary and results tabulated to see whether or not the average length of treatment of the cases was affected; whether the pathologic conditions averaged as severe as in cases in which emetin was not used; whether recurrences were as likely, etc., etc.

So the question is not a simple one to be lightly dismissed. I hesitate to discuss this question of treatment, but being cognizant of the possibilities for evil of some of the amœba elsewhere, and knowing the specific action of emetin toward this organism, I can not but cry a warning for the possibility of dangers of over-enthusiasm on the one hand, and the unfair discrediting of a possible useful drug on the other.— Extract from article in August *Summary.*

